# Fathers' Experience of Their Child's Paediatric Brain Tumour

**DOI:** 10.1002/cnr2.70061

**Published:** 2024-12-03

**Authors:** Florence Labrell, Christine Hassler, Christelle Dufour, Jacques Grill, Hugo Câmara‐Costa

**Affiliations:** ^1^ Université Paris‐Saclay, Université Paris‐SUD, UVSQ, CESP, INSERM Paris France; ^2^ INSEI (National Institute for Inclusive Education) Suresnes France; ^3^ Department of Paediatric and Adolescent Oncology Gustave Roussy Villejuif France; ^4^ Rehabilitation Department for Children With Acquired Neurological Injury, Saint Maurice Hospitals Saint Maurice France; ^5^ Sorbonne Université, CNRS, INSERM, Laboratoire d'Imagerie Biomédicale, LIB Paris France; ^6^ Sorbonne Université, GRC 24 Handicap Moteur et Cognitif et Réadaptation (HaMCRe) Paris France

**Keywords:** paediatric brain tumours, paternal experiences, qualitative analysis, representations of the child

## Abstract

**Background:**

This exploratory study explores the impact of paediatric brain cancer on the experiences of the fathers from the time of diagnosis, while most studies have focused on the mothers.

**Methods:**

The content of interviews conducted with six fathers of children who had brain tumours at the age of approximately 10 years was analysed using a qualitative methodology, following the COREQ guidelines.

**Results:**

The fathers first talked about their feelings about the way the brain tumour affected their child and how he/she coped with the illness and treatments, and they also described the difficulties encountered. These French fathers likewise talked about their rights, their need to be listened to, and their wish to remain close to their child during treatment.

**Conclusion:**

Improving communication with fathers during care and medical visits could help promote more balanced support for both parents in the vulnerable period of serious paediatric illnesses such as brain tumours.

The role of parents in the recovery of a child or an adolescent with cancer has been widely explored [1, 2, 3]. Parents need to fulfil this role despite the trauma of their own experience of the illness, and they need to develop ways of coping with it themselves [2, 3, 4, 5, 6, 7, 8]. In addition, they have to do so from the very moment their child is diagnosed and sometimes over several years in case of relapse [9] or death. Results on the subject of gender differences in the traumatic experiences of parents have yielded contrasted results [10, 11, 12]. Nonetheless, fathers seem more likely to develop long‐term anxiety symptoms than mothers. It has also been shown that fathers' adjustment to their child's life‐threatening illness is less effective than that of mothers. For instance, a quantitative study found that in the year following diagnosis, fathers were more likely to exhibit depressive symptoms than mothers [13]. This particular finding might be related to the fact that fathers have fewer opportunities to express their emotions in the painful period of diagnosis, as they tend to be socially encouraged to resume full‐time work and to provide financial support for the family [13]. It can be added that successful coping by the two parents has a protective effect against marital separation, which is a frequent consequence of chronic, severe paediatric illnesses [14, 15].

Nonetheless, studies on parents whose children have cancer have mostly concerned mothers, although a literature review noted that research among fathers was increasing [6]. A recent review on participation in psychosocial research in oncology reported several reasons that could explain why fathers were less likely to agree to participate than mothers: mothers tend to be more involved in childcare and the recruitment of fathers is more complex except when they are recruited in the work‐place [16].

It therefore appeared to us that there was a need to consider fathers' experiences of paediatric cancers, such as paediatric brain tumours (PBTs), areas where these experiences have been very little studied [7] unlike maternal experiences [4, 17]. Indeed, brain tumours are the most frequent solid paediatric tumours [18], affecting 500 children per year in France [19] and causing more cognitive impairment [20] and academic difficulties than other cancers [21, 22]. Despite advances in treatment, they remain the most fatal tumour type among children [18].

Results from the few qualitative studies on paternal experiences of paediatric cancer have shown that the protective factors that help fathers cope include relationships with and support from the medical team [23, 24] alongside support in their work‐place [25]. Qualitative analyses of focus groups with fathers have shown that they find it difficult to simultaneously maintain their role of breadwinners and express their emotions openly (“crying in secret” [23]). The only recent qualitative study on fathers dealing with PBTs also described the value of listening to their expressed needs in terms of their roles: “being part of a parent team, being a father, a supporter, a provider, being present, and being a protector” [26] (p. 1). Alongside, a French study noted that in cases of bad news in paediatric oncology, mothers were more likely to receive information from the medical team than fathers, and they also tended to pass on less information to the fathers, to spare them [27]. However, no French study has yet examined fathers' experiences of PBTs.

## 1 Aims of the Study

While much research on parental experiences of paediatric cancer assumes that the two parents have the same experience, some studies have suggested that interventions should consider both the specificities and the similarities of the two parents in order to tailor communication and psychosocial care to their respective needs [6, 23, 26]. However, fathers' experiences of PBTs have been explored significantly less than mothers' experiences. A recent systematic review of qualitative research on family experiences of PBTs showed that only one study focused exclusively on fathers' experiences [28], despite the fact that a father's contribution to their child's development is just as important [29]. The aim of this exploratory study was therefore to investigate how French fathers experience PBTs, as there is currently no research on this topic within this population.

## 2 Methods

### 2.1 Participants

This qualitative exploratory study on paternal experiences of PBTs recruited eight fathers who lived in France, spoke French, and were able to read the written consent and participate in the interviews. All fathers in the sample were of French nationality. Six participated in the study, while two finally declined for reasons of personal availability. Table 1 summarizes the sociodemographic data.

**TABLE 1:** Social and demographic characteristics of the study sample.

Child	
Gender	
Boys	3 (50%)
Girls	3 (50%)
Mean age (in years)	10.5 years Range [7–14]
Time since diagnosis	6 years and 7 months Range [4–8]

Father	
Mean age	39.4 years Range [36.8–43.8]
Ethnicity	
Caucasian	6 (100%)
Marital status	
Married/Living with partner	4 (66.6%)
Single	2 (33.3%)
Educational level	
Below *Baccalauréat* level^a^	3 (50%)
At least some higher education	3 (50%)

^a^ The *Baccalauréat* examination is a prerequisite for higher education access in France.

The social and demographic data showed that the fathers in the sample were about 40 years old, mostly living with a partner, with similar levels of education and qualification. The mean age of the children with brain tumours was 10 years, half were girls, none were an only child, all of them had been diagnosed more than 6 years previously, and none was currently undergoing treatment. They could thus be described as cancer survivors.

The population was drawn from an earlier study aiming to validate a French version of an assessment instrument measuring parental stress in a sample of mothers whose children had developed a brain tumour [4]. The file submitted to the ethics review board for the first study allowed for the possibility of subsequent follow‐up requests. Of the 35 families included in the larger study, only eight fathers responded and participated on a voluntary basis. This second retrospective study focused on fathers' experiences of PBTs, interviewing the fathers from the same families whose children had been treated in a hospital near Paris specialized in the treatment of paediatric cancers. The sample consisted of six fathers who were available at the time and gave their informed consent. The inclusion criteria were that at least 4 years had elapsed since the child's diagnosis and that the fathers understood, spoke, and read French.

### 2.2 Ethics Statement

The present study (SPELECA project) was approved by the ethics review board from the Institut Curie in Paris (Cancéropôle IdF, call for proposals Emergence, principal investigator F. Labrell) in January 2016 and by the ethics committees of the participating hospital in the Parisian area, where the children had received treatment (Gustave Roussy and St Maurice Hospitals).

### 2.3 Data Collection

The data collection and analysis of the interviews implemented qualitative methods, which have shown their relevance in the few studies on paternal experiences of paediatric cancer [24, 26, 30].

The fathers were invited to attend a semi‐structured interview in the relevant hospital on the day of one of their child's medical follow‐up visits, in the period May–July 2022. The fathers were told that the aim of the study was to explore their experiences of paediatric cancer. The interviews were recorded after the fathers had received information about the study and had provided personal data such as age, occupation, and marital status. The researcher, who was a psychologist, used an interview guide based on previous studies [24, 26, 30], focused on the impact of the illness on the family and on the fathers' experiences since at the end of the interview (average duration: 30 min), the researcher gave the fathers the opportunity to provide any additional information they considered relevant.

### 2.4 Data Analysis

Firstly, the interview data was transcribed in French word for word. Four researchers (A, B, CM, CT) analysed the transcripts using thematic analysis complemented by discourse analysis [31] using qualitative analysis software (NVivo, version 11), so as to facilitate qualitative analyses implementing an inductive procedure. Then, A and B independently defined themes and labels, confronted their findings and agreed on a tree plot of nodes. All the researchers coded the interview transcripts separately to triangulate the themes derived from the data and to ensure that the attribution of quotes to nodes was sufficiently relevant. At the end of the analysis, each corpus could thus be characterized by a number of key phrases corresponding to the themes and sub‐themes. The researchers agreed on a tree plot of nodes for analysis. A sample of 717 key phrases from the 6 interviews was coded in terms of 4 main themes and 9 subthemes from the codebook (see Table 2).

**TABLE 2:** Themes and subthemes of the codebook and examples.

Themes	Subthemes	Examples
Father's feelings	Feelings about the illness	*It was really complicated, I really feared the worst (1)* *In periods when he was hospitalised, we found accommodation nearby*
	Support and needs	
	Coping	*I joined in the combat, because there was no other choice (2)*
The sick child	Courage	*She showed nothing, not even her vulnerability. She is incredibly strong (3)*
	Maturity	*He fought, he persevered, he never collapsed (4)*
Temporality	Diagnosis	*There really is a before and after (3)*
	Burden over time	*We don't know yet, for the moment we are managing one day to the next (2)*
	The future	*In 6 months she may no longer be with us—I don't want to bother her with that (3)*
The place of the father	Society	*People think that Mum feeds and Dad protects (6)*
	Inequalities	*I think there are great inequalities between father and mother* *In case of serious illness for the child, what place for the father? It's a taboo subject (5)*

The themes and sub‐themes were discussed by all the researchers. The study followed the COREQ guidelines (the Consolidated Criteria for Reporting Qualitative Research) [32].

## 3 Results

The results of the qualitative analysis of the interviews with the fathers^1^ are presented according to themes.

### 3.1 Father's Feelings

One of the major themes concerned paternal feelings about their child's illness, which the fathers talked about first in the interview: “*I have never been so frightened in my life* (at the time of the diagnosis), *today, paradoxically, I am no longer afraid of much else*” (1). Despite some variability in the tendency to inhibit expressions of distress, each father was able to express his pain, even several years after the diagnosis and in the absence of ongoing treatment. One of the subthemes concerned support, mainly from parent associations providing help for sick children and their families; the fathers talked about their own needs for individual support, particularly from friends. Several fathers in our study mentioned that they would have liked to have someone to talk to, someone to listen to them, especially during the waiting periods at the hospital, as a preferred alternative to online support. Regarding needs and coping, one father mentioned that he appreciated being able to release tension through physical activity: “*Instead of going out to eat at lunchtime, I would go out and play sports and it made me feel better*” (2). At the same time, a lexical analysis of the transcripts showed that the notion of a “combat” was often mentioned by the fathers in relation to the course and seriousness of the disease: “*we are fighting for her to be still here in 10 years' time*” (3).

### 3.2 The Sick Child

Another major theme concerned the child with cancer, not only in terms of illness, care, and management but also in relation to the child's resilience and courage: “*She's able to withstand a lumbar puncture and a spinal puncture on the same day by gritting her teeth*” (3). In addition, some fathers mentioned their sick child's particular maturity, surpassing for example that of a healthy older sibling: “*It has given her a maturity that we do not see in her sister who is 4 years older*” (1). The frequency of the fathers' references to their child was notable because the instructions, before these semi‐structured interviews were carried out, did not mention the child, but only the fathers' experiences, which was the aim of the interview.

### 3.3 Temporality

The temporal trajectory of the PBT was another theme indirectly reported by the fathers, whose statements contained several elements relating to temporality. We identified three sub‐themes relating to time. Firstly, the fathers mentioned the moment of diagnosis, a time pervaded with anxiety. For instance, one of them talked about the moment “*when the* EMS (Emergency Medical Service) *arrived at the house…I am a fairly reactive person*” (4). Secondly, they discussed the burden of the illness over time, which came with a certain gradual awareness: “*It's not when you learn that your child has a brain tumour that it's the most difficult, there are ups and downs*” (4). Finally, the fathers mentioned the child's future and development, in relation to the illness: “*If it turns out that in 6 months it's no longer there*…” (3), “*for the moment, it's more or less okay…after that we will wait and see how it all evolves*” (1).

### 3.4 The Place of the Father

The place of the father in case of PBT also emerged as a theme, with a subtheme of fathers as full stakeholders in the process of cure of their child. On the one hand, society was described as supportive towards parents of children with cancer: “*We get a lot of help…when you say*” “*I have a child with cancer, he's on chemo*” (5). But some fathers also considered society as a source of inequalities for fathers compared to mothers: “*There are very big inequalities with respect to men… and I think women and society don't even see it*” (5), “*I've been through two divorces…and fathers can have that kind of feeling that they're the least important part of the family*” (1).

## 4 Discussion

The purpose of this section is to interpret the four dimensions that emerged from the fathers' interviews highlighting their strengths in relation to previous research. We also explore similarities with those identified in the only other study on this topic to date [26].

### 4.1 Fathers' Experiences and Representations of Their Sick Child

The qualitative analysis of fathers' experiences of PBTs highlights their feelings about this serious illness affecting their child. Our results are congruent with previous studies showing that fathers tend to develop anxiety symptoms at a greater distance from the diagnosis than mothers [13]. We could also hypothesize that these feelings experienced by the fathers were specifically linked to the uncertainty of PBTs—uncertainty interpreted as a danger, underpinning their psychological distress. This distress could in turn hinder the child's treatment [33]. The theory of uncertainty in relation to illness “explains how people give meaning to the advent of illness, where uncertainty indicates the absence of meaning” [34] (p. 225).

The fathers' descriptions of their sick child did not just focus on their own distress or needs, they also referred to the child's courage, resilience, and increased maturity. Therefore, even if the notion of the fight against cancer was mentioned by some fathers, they did not only describe their sick child as a fragile being.

### 4.2 Fathers' Ways of Coping and Needs

The French fathers in our study talked about their own needs for individual support from friends. Nonetheless, a review of the qualitative literature on parental experiences of children with cancer showed that when fathers asked male friends for support, these men did not know how to help them [35]. The authors therefore suggested that meetings should be set up in hospitals where fathers of children with cancer could talk and help each other, in the form of support groups. As far as we know, this type of support is still not very frequent in French hospitals, as reported by the fathers in our study. Similarly, a recent literature review showed that psychosocial interventions offered to families of children with cancer, in particular therapies intended to alleviate maternal distress, have a significant effect on parental empowerment [36]. On the other hand, these French fathers were not necessarily willing to seek out or participate in, online support, as recommended by a recent review [37].

### 4.3 Temporal Issues

Our results showed that the theme of time was very common when fathers talked about their experiences of the illness trajectory. Indeed, paediatric cancers, particularly certain brain tumours (such as medulloblastoma), tend to become chronic and generate parental stress, from the time of diagnosis to the alternation between remissions and relapses [38, 39, 40, 41]. As our small sample consisted of fathers whose children had been diagnosed earlier and were not currently receiving treatment, we can assume that the reference to time was not restricted to the recent history of their child's illness. Thus, references to time expressed by the fathers seem to be linked to the progression of the disease and concerns about the children's future were related to the course of the disease and to questions about the children's future.

Our results have also highlighted new issues related to social and cultural representations.

### 4.4 How do French Fathers See Their Inclusion as Stakeholders in the Process of Cure for the Child?

Results about the way the French fathers felt involved in the care of PBTs provide information about the social representations of the paternal role in a context of serious childhood illness. These paternal experiences in cases of PBTs highlight the traditional roles played by fathers and mothers when dealing with their child's illness. This traditional division of roles, which is frequent in cases of life‐threatening illnesses [42], corresponds to societal expectations based on social and cultural representations. This question is in line with results on how some American fathers define themselves, not only as “rocks” to lean on, but also as parents close to their child, and as partners, wishing to be more present at the hospital with their child rather than merely providing financial resources [26]. Similar to fathers in the USA, French fathers expressed the desire to be considered as active participants in their child's care, and wishing to be closer to their child, especially when he/she is seriously ill [43]. However, given the small size of our sample, the results of our study need to be verified on a larger scale.

## 5 Limitations

One of the limitations of this exploratory study was the small, homogeneous study sample. In this qualitative study exploring the experiences of French fathers dealing with their child's cancer, our sample lacked ethnic diversity, a recognized factor significantly affecting parental experiences [35]. Consequently, the themes highlighted in the fathers' discourse probably did not comprehensively reflect the full extent of paternal experiences related to this specific paediatric illness. In addition, the results obtained from the current study were based on a relatively limited sample of fathers, and additional data from a more diverse sample of fathers is needed to verify these findings.

Further studies are necessary. For instance, additional qualitative studies using the same interview guide and also interviewing mothers of children with cancer would enable an exploration of their perceptions of the fathers' experiences. These studies might follow the results from British mothers who considered that fathers often choose to invest in the professional sphere as a strategy to avoid this difficult situation [17]. Comparing fathers' and mothers' experiences could help identify their respective needs, particularly concerning their inclusion in caregiving. Finally, future studies should consider the time since diagnosis when exploring the themes associated with parental stress following PBT, whereas in the present study, the interviews were conducted at a single timepoint, which varied in terms of the time lapse since the child's diagnosis.

## 6 Conclusion

Beyond the general findings on paternal experiences after paediatric cancer, this exploratory study, based on a rigorous application of a qualitative methodology, highlights the unique characteristics of paternal experiences throughout the trajectory of PBTs. French fathers expressed their desire to be included as full stakeholders in their child's treatment process. While future studies are needed, the current results are noteworthy for improving and balancing care between both parents during periods of intense stress induced by a potentially life‐threatening illness like PBTs. Establishing psychological counselling for fathers throughout the care pathway French paediatric oncology departments would be beneficial for families affected by PBT.

## Author Contributions

**Florence Labrell:** conceptualization, methodology, data curation, writing – original draft. **Christine Hassler:** conceptualization, methodology, data curation. **Christelle Dufour:** conceptualization, resources. **Jacques Grill:** conceptualization, resources. **Hugo Câmara‐Costa:** conceptualization, methodology, data curation.

## Conflicts of Interest

The authors declare no conflicts of interest.

## Data Availability

Research data are not shared.

## Endnotes

^1^
The father who spoke is anonymized by the number in brackets at the end of each quotation.
